# The impact of transition shock on moral sensitivity among nursing interns and its mechanism: a cross-sectional study

**DOI:** 10.3389/fmed.2026.1773512

**Published:** 2026-02-26

**Authors:** XuYan Liu, RenLong Liang, Jijun Wu, Xiaoli Zhong, YiWei Li, Yin Yuan, Heng Ding, Tingting Cai, Tingting Ruan

**Affiliations:** 1 Department of Nursing, Deyang People’s Hospital, Deyang, Sichuan, China; 2 Department of Neurology, Deyang Hospital Affiliated Hospital of Chengdu University of Traditional Chinese Medicine, Deyang, Sichuan, China; 3 Department of Gynecology, Deyang People’s Hospital, Deyang, Sichuan, China

**Keywords:** calling, moderated mediation, moral sensitivity, nursing interns, self-compassion, transition shock

## Abstract

**Background:**

Nursing interns experience transition shock when shifting from theory to practice. This shock continuously depletes their psychological resources, leading to self-doubt. Consequently, their attention to patients’ ethical situations is diverted, and their moral sensitivity decreases. Therefore, it is necessary to explore the underlying mechanisms and propose strategies to mitigate the impact of transition shock on moral sensitivity.

**Methods:**

An online questionnaire survey was conducted among participants from June to December 2024. The survey included questions about general demographics, the Transition Shock Scale for Undergraduate Nursing Students, the Self-Compassion Scale, the Chinese Revised Version of the Moral Sensitivity Questionnaire, and the Chinese Calling Scale. Spearman correlation analysis was used to clarify the correlations among variables. Models 4 and 7 in the PROCESS v4.2 macro (a plug-in for SPSS 27) were used to verify the mediating and moderating effects, respectively. RStudio was used to plot the simple slope graph.

**Results:**

Among nursing interns, transition shock was a key predictor of moral sensitivity, with a significant negative correlation (*r* = −0.320, *p* < 0.01). Self-compassion partially mediated the relationship between transition shock and moral sensitivity, accounting for 42.59% of the total effect. Additionally, calling attenuated the negative predictive effect of transition shock on self-compassion. As calling increased, the mediating effect of self-compassion gradually decreased.

**Conclusion:**

Educational institutions and internship hospitals should strengthen the cultivation of self-compassion and calling among nursing interns. This can alleviate transition shock, promote the development of moral sensitivity, and lay the foundation for them to grow into qualified, empathetic, and ethically literate nurses.

## 1 Background

**Figure 1:**
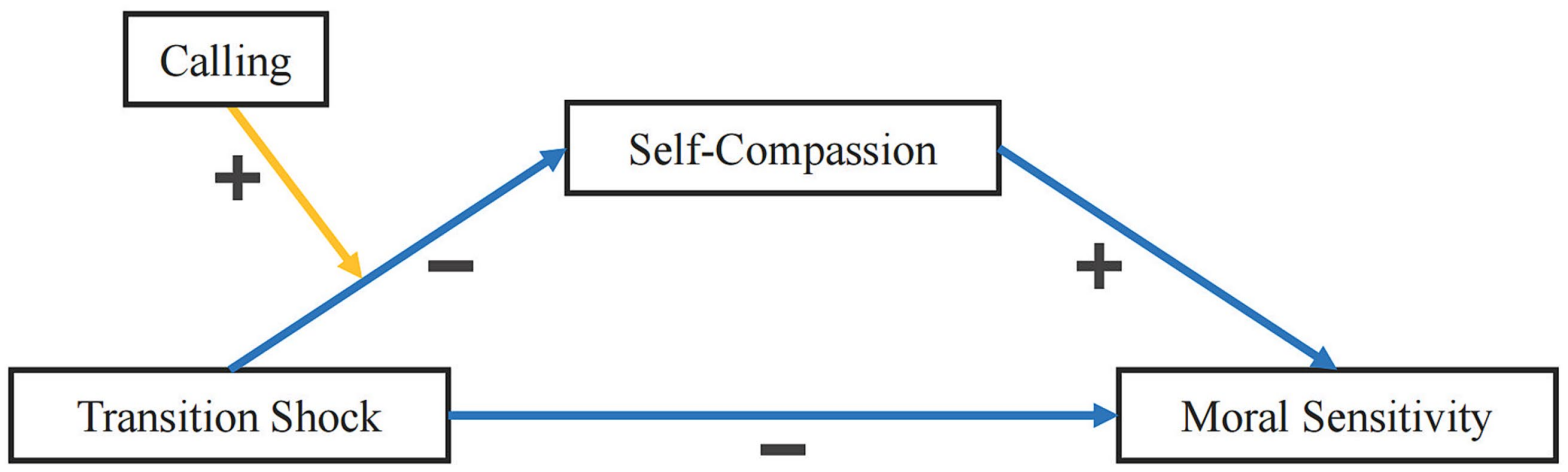
Hypothesis-based structural equation diagram. n.s. = not significant; critical point = threshold.

Nursing interns are in a critical transitional period, shifting from “students” to “nurses.” Due to changes in their roles, interpersonal relationships, responsibilities, knowledge, and skills, they may experience feelings of confusion, perplexity, doubt, and ambiguous self-positioning (1). Moral sensitivity refers to nurses’ continuous attention to and perception of patients’ ethical needs. As the core starting point of nursing ethical decision-making (2), the level of moral sensitivity is directly related to the ethical quality of nursing services (3). Current research has focused on the impact of transition shock on nurses’ morality-related literacy (4–6) and the positive influence of calling on ethical practice in the nursing profession (7). However, deficiencies remain. First, the mechanism by which transition shock affects nursing interns’ moral sensitivity is unclear. Second, the moderating role of calling is often explored in isolation (8), and there is a lack of integrated analysis on how it synergizes with the mediating effect of self-compassion to regulate the impact pathway of transition shock on moral sensitivity. This study constructs and verifies a moderated mediation model (see Figure 1) to identify key influencing factors and action pathways. This will facilitate the development of targeted intervention strategies that alleviate transition pressure and enhance moral sensitivity and ethical practice capabilities among nursing interns.

As reserve forces of the medical team, the transition shock experienced by nursing interns has become a key factor affecting their professional development and ethical practice (9). From a theoretical perspective, Duchscher’s Transition Shock Theory (10) suggests that nursing interns must progress through the stages of “role expectation,” “role practice,” and “role identity.” Transition shock is essentially role conflict and adaptation pressure triggered by insufficient environmental support (e.g., inadequate preceptorship guidance) and a mismatch between abilities and needs (e.g., difficulty in translating theoretical knowledge into practical operational skills). According to Hobfoll’s Conservation of Resources Theory (COR), this type of shock constitutes a loss of psychological resources, consuming the energy nursing interns use for emotional regulation and cognitive focus. When transition shock leads to the exhaustion of these resources, nursing interns become prone to cognitive biases that prioritize their own dilemmas. This diverts their attention from patients’ ethical situations, thereby reducing their moral sensitivity (11). From an empirical research perspective, although existing studies have not directly examined the relationship between the two, relevant evidence has provided support. Studies have found that transition shock can lead to job burnout (12), increase turnover intention (13), and cause cognitive biases and impulsive decision-making (14), resulting in errors. These findings suggest that transition shock continuously consumes nursing interns’ psychological resources and occupies their attention, thus impairing their ability to perceive ethical issues. Thus, the following hypothesis is proposed:

*Hypothesis 1:* Transition shock has a significant negative impact on moral sensitivity among nursing interns.

As a positive psychological resource, self-compassion emphasizes an individual’s kind acceptance of oneself, mindful awareness of pain, and recognition that difficulties are shared human experiences (15). This concept aligns perfectly with the psychological adjustment needs of nursing interns in the context of transition shock. From a theoretical perspective, according to COR (16), the depletion of psychological resources caused by transition shock directly weakens the level of self-compassion. Further deduced from Self-Compassion Theory (17), a high level of self-compassion can reduce self-criticism and alleviate emotional exhaustion (18), preserving the psychological energy for individuals to focus on external ethical situations (19). In other words, when nursing interns possess good self-compassion abilities, even in the face of transition shock, they can avoid being distracted by self-negation and are more likely to perceive patients’ ethical needs—for example, consciously protecting patients’ privacy during daily nursing care.

In empirical studies, Miller et al. (20) found that mindfulness and kindness have a positive impact on moral sensitivity. Kotera et al. (21) identified self-compassion as a key explanatory variable for mental health, noting a significant relationship between self-compassion and moral judgment, with self-compassion contributing to reducing negative emotions and enhancing mental health. Kroshus et al. (22) surveyed 5,509 first-year college students and demonstrated that self-compassion plays a vital role in alleviating transition shock-related stress and improving well-being among undergraduates. These findings suggest that self-compassion may act as a mediating role between transition shock and ethics-related literacy. Integrating theoretical logic and gaps in empirical research, the following research hypotheses are proposed:

*Hypothesis 2:* Transition shock has a significant negative impact on self-compassion among nursing interns.

*Hypothesis 3:* Self-compassion has a significant positive impact on moral sensitivity among nursing interns.

*Hypothesis 4:* Self-compassion mediates the relationship between transition shock and moral sensitivity among nursing interns.

Calling is defined as “the integration of occupation and life meaning” (23), manifested as internal recognition of the value of the nursing profession and willingness to dedicate oneself to it long-term. Its characteristic of “meaning reconstruction” makes it a potential variable for moderating the impact of transition shock (24). Nursing interns with high calling tend to interpret transition shock as a necessary test for professional growth rather than a mere stressor (25). This meaning reconstruction can reduce the consumption of self-compassion resources caused by shock (26). Specifically, when facing transition conflicts, interns with high calling are more likely to accept their own shortcomings and avoid falling into self-criticism due to short-term difficulties, thereby maintaining their level of self-compassion.

In terms of empirical research, Xin et al. (13) studied 739 newly graduated nurses and found that transition shock can reduce the level of calling, thereby decreasing retention intention. Li et al. (4) investigated 506 healthcare professionals and showed that moral injury has a negative impact on calling, while self-compassion and ethical leadership can buffer this negative effect. These findings indicate a potential internal connection between transition shock, self-compassion, and calling. Based on theoretical characteristics and empirical clues, the fifth research hypothesis is proposed:

*Hypothesis 5:* Calling moderates the relationship between transition shock and self-compassion among nursing interns; specifically, the higher the calling, the weaker the negative impact of transition shock on self-compassion.

## 2 Methods

### 2.1 Study design

A descriptive cross-sectional correlational study was adopted, following the STROBE guidelines.

### 2.2 Sample size calculation

G*Power 3.1 software was used to calculate the sample size (27). According to Cohen’s criteria (28), the significance level was set at 0.05, statistical power at 95%, and the effect size at medium (*f*^2^ = 0.15). There were 18 predictor variables in this study. Based on these parameters, G*Power calculated that 213 samples were needed. Considering the complexity of the structural equation model, we aimed to recruit additional samples to improve stability, reduce multicollinearity, and handle missing data. Finally, 350 nursing interns were invited to participate.

### 2.3 Sample and setting

From June to December 2024, a total of 350 nursing interns were recruited from 6 hospitals in Sichuan Province, China, using convenience sampling for an online questionnaire survey. Among them, 12 questionnaires were excluded due to incomplete responses, 18 for illogical responses, and 11 for meeting the exclusion criteria after verification. Finally, 309 valid questionnaires were obtained, with an effective recovery rate of 88.29%.

The inclusion criteria of this study were as follows: being undergraduate or junior college nursing students who had completed theoretical courses; having no prior nursing practice experience before the internship; interning in clinical frontline departments; having an internship duration of ≥ 1 month and ≤ 6 months in clinical departments; and providing informed consent and cooperating voluntarily with the survey.

The exclusion criteria of this study were as follows: being refresher nurses, standardized training nurses, or regular nurses; interning in non-clinical frontline departments; having an internship interruption of more than 2 weeks; and participating involuntarily.

### 2.4 Research tools

#### 2.4.1 General information survey form

The questionnaire was designed based on a summary of previous literature and expert consultation. It includes the following dimensions: (i) Basic demographic characteristics: Gender, age, place of origin, whether being an only child, whether planning to engage in nursing after graduation, and whether they have family support for a nursing career. Nursing interns with strong family support are more likely to receive emotional comfort when facing role conflicts or work pressure, thereby alleviating the confusion and helplessness caused by transition shock. (ii) Academic-related attributes: Education level, whether having received ethics-related course training at school, and attitude toward the nursing profession. (iii) Internship scenario information: hospital grade, whether having received pre-internship skill training, internship department, and internship duration.

#### 2.4.2 Transition shock scale for undergraduate nursing students

Kim et al. (29) developed the Undergraduate Nursing Transition Shock Scale (UNTSS) in 2018 based on the Transition Shock Theory. This scale has been applied in multiple international studies (30, 31) and exhibited good reliability and validity, making it a mature tool for assessing transition shock among undergraduate nursing interns. Huang et al. (32) localized the scale to develop the Chinese version of the UNTSS, which consists of 6 dimensions and 18 items. The dimensions include conflict between theory and practice, excessive internship workload, lack of social support, tense interpersonal relationships, confusion about nursing professional values, and inconsistency between clinical internship and personal life. The scale uses a 4-point Likert scale, ranging from “strongly disagree” to “strongly agree,” scored on a 1–4 scale. The total score ranges from 18 to 72, with higher scores indicating more severe transition shock. The Cronbach’s *α* coefficient of the total scale is 0.912, and the content validity index is 0.987 (32). To expand the application scope of this Chinese version, Weng et al. (33) applied it to junior college interns and verified its reliability and validity. The results showed that the scale had high internal consistency reliability, with a Cronbach’s *α* coefficient of 0.925 and an intraclass correlation coefficient of 0.908. These findings indicate that the Chinese version of UNTSS can be used to measure the degree of transition shock among both undergraduate and junior college nursing students.

#### 2.4.3 Self-compassion scale

The level of self-compassion was assessed using the Self-Compassion Scale, which was developed by Neff (34) and subsequently localized and validated in Chinese by Chen et al. (35). This scale comprises 6 dimensions (self-judgment, self-kindness, common humanity, isolation, mindfulness, and over-identification) with a total of 26 items. A 5-point Likert scale was adopted, ranging from “strongly disagree” to “strongly agree,” with scores assigned from 1 to 5 accordingly. Notably, three dimensions—self-judgment, isolation, and over-identification—were reverse coded. The total score ranges from 26 to 130, with higher scores indicating a higher level of self-compassion. The Cronbach’s *α* coefficient of the scale was reported as 0.84 in the validation study by Chen et al. (35), and in the present study, the Cronbach’s α coefficient of the scale was 0.817.

#### 2.4.4 Chinese version of the moral sensitivity questionnaire-revised

Lutzen et al. (36) revised the Moral Sensitivity Questionnaire (MSQ) in 2006. The revised version (MSQ-R) has been widely used in the nursing field (37, 38). Huang et al. (39) translated the MSQ-R into a Chinese version (MSQ-R-CV) translated and culturally adapted for the Chinese context. Studies have shown that the MSQ-R-CV is more linguistically and culturally appropriate for Chinese nurses and has good reliability and validity (40). The Cronbach’s *α* coefficient of this scale was 0.820 (39). A 6-point Likert scale was adopted for the MSQ-R-CV, ranging from “I strongly disagree” to “I strongly agree,” with scores from 1 to 6, respectively. The total score ranges from 9 to 54, with higher scores indicating higher moral sensitivity. Specifically, scores < 32 indicate low sensitivity, 32–43 indicate moderate sensitivity, and scores > 43 indicate high sensitivity. In the present study, the Cronbach’s *α* coefficient of the scale was 0.777.

#### 2.4.5 Chinese calling scale

The Calling Scale was developed by Zhang et al. (41), consisting of 3 dimensions (guidance, altruistic contribution, and proactive initiative) with a total of 10 items. A 5-point Likert scale was used for scoring, ranging from “completely inconsistent” to “completely consistent,” with scores assigned 1 to 5, respectively. The total score ranges from 10 to 50, which can be divided into 5 levels: 10–18 (low level), 19–26 (low-moderate level), 27–34 (moderate level), 35–42 (moderate-high level), and 43–50 (high level). Higher scores indicate stronger individual calling. The overall Cronbach’s *α* coefficient of the scale was 0.89 when validated in groups across multiple different occupations (41). In the present study, the Cronbach’s α coefficient of the scale was 0.911.

### 2.5 Data collection and quality control methods

Members of the research team contacted nursing administrators at the participating hospitals to assist with questionnaire distribution. The survey was administered via using “Wenjuanxing” (a widely used online questionnaire platform in China) via WeChat mini-program, a widely used online questionnaire platform in China. At the start of the questionnaire, participants were provided with the study purpose, study procedures, and a detailed description of the inclusion and exclusion criteria to obtain valid informed consent and ensure the quality of raw data. We explicitly stated that no sensitive information (e.g., names, contact details, or home addresses) would be collected, and that all collected data would be used exclusively for research purposes. Each WeChat account was restricted to one submission, and questionnaires could only be submitted after a minimum completion time of 10 min. Two researchers independently reviewed all data entries, and questionnaires with illogical responses, failure to meet the inclusion/exclusion criteria, or incomplete responses were excluded.

### 2.6 Statistical analyses

SPSS 27.0 software was used for all statistical analyses: general information was summarized using frequencies and percentages; for other variables, the Shapiro–Wilk test was applied to assess normality of the data. The results indicated that none of the variables followed a normal distribution (all *p* < 0.05), so medians and interquartile ranges (P25, P75) were used for descriptive statistics. Correlations: Spearman correlation analysis for transition shock, self-compassion, moral sensitivity, and calling; Common method bias: assessed using Harman’s single-factor test; Mediating effect: verified by Model 4 in Hayes’ Process v4.2 macro program; Moderating effect: verified by Model 7 in Process v4.2, with simple slope analysis; Bootstrap method (*n* = 5,000) was used to test mediating and moderated mediating effects, with 95% confidence intervals (CIs). Effects were considered significant if 95% CI did not include zero. The significance level was *α* = 0.05. Rstudio software was used to draw simple slope plots.

### 2.7 Ethics statement

This study followed the ethical standards of the Declaration of Helsinki and was approved by the Ethics Committee of Deyang People’s Hospital (Approval No.: 2024-04-072-K01). Informed consent was obtained from all respondents. They were informed that personal information would be anonymized and no sensitive information would be collected. Respondents could refuse to participate or withdraw at any time.

## 3 Results

### 3.1 General information of the surveyed nurses

A total of 309 valid questionnaires were collected and included in the analysis. The general demographic characteristics of the nursing intern participants are detailed in Table 1. Specifically, 81.9% of the respondents were female, and 62.8% were aged between 21 and 23 years. Among them, 65.0% were only children (had no siblings), 60.8% were rural residents, and 89.3% had family support for working in nursing after graduation, while 17.8% were unwilling to work in the nursing profession post-graduation. The proportion of respondents with a bachelor’s degree was higher than that of those with a junior college degree. Additionally, 79.9% of the interns reported that their schools had provided systematic ethics-related training, and 84.8% indicated they had not received standardized pre-internship skills training. A total of 69.3% interned in tertiary hospitals, 56.0% expressed a liking for the nursing profession, and 89.6% had been interns for 1 to 5 months.

**Table 1:** General demographic characteristics of respondents (*n* = 309).

Variables	Category	Frequency	Percentage (%)
Gender	Male	56	18.1
	Female	253	81.9
Age (years)	18–20	89	28.8
	21–23	194	62.8
	>23	26	8.4
Number of siblings	0	201	65.0
	1	79	25.6
	2	23	7.4
	≥3	6	1.9
Place of origin	Urban	121	39.2
	Rural	188	60.8
Family support for nursing	Yes	276	89.3
	No	33	10.7
Intention to engage in nursing after graduation	Yes	254	82.2
	No	55	17.8
Education level	Undergraduate	179	57.9
	College	130	42.1
School offered ethics-related courses	Yes	247	79.9
	No	62	20.1
Hospital provided pre-internship skill training	Yes	47	15.2
	No	262	84.8
Grade of internship institution	Grade II	95	30.7
	Grade III	214	69.3
Internship department	Internal medicine	111	35.9
	Surgery	103	33.3
	Emergency department	27	8.7
	Others	48	15.5
	ICU	20	6.5
Attitude toward the nursing profession	Like	173	56.0
	Dislike	63	20.4
	Not certain	73	23.6
Internship duration (months)	1 < duration < 3	175	56.6
	3 ≤ duration < 5	102	33.0
	5 ≤ duration ≤ 6	32	10.4

### 3.2 Common method bias test

Harman’s single-factor test was performed to assess common method bias among all variables. The results showed that a total of 18 factors had eigenvalues greater than 1, and the variance explained by the first common factor was 14.57%, which was below than the critical threshold of 40%. This suggests that no substantial common method bias was present in this study.

### 3.3 Correlation analysis between variables

The normality test indicated that the scores of all variables did not follow a normal distribution. Therefore, medians and interquartile ranges (P25, P75) were used for descriptive statistics, and Spearman correlation analysis was applied to examine the correlations among variables. The results showed that: Transition shock was significantly negatively correlated with moral sensitivity (*r* = −0.320, *p* < 0.01); Transition shock was significantly negatively correlated with self-compassion (*r* = −0.386, *p* < 0.01); self-compassion was significantly positively correlated with moral sensitivity (*r* = 0.549, *p* < 0.01); Calling was significantly negatively correlated with transition shock (*r* = −0.150, *p* < 0.01), significantly positively correlated with self-compassion (*r* = 0.283, *p* < 0.01), and significantly positively correlated with moral sensitivity (*r* = 0.176, *p* < 0.01). See Table 2 for details.

**Table 2:** Correlation analysis between variables (*n* = 309).

Variable	Transition shock	Self-compassion	Moral sensitivity	Calling
Transition shock	1	—	—	—
Self-compassion	−0.386^**^	1	—	—
Moral sensitivity	−0.320^**^	0.549^**^	1	—
Calling	−0.150**	0.283^**^	0.176^**^	1
M	50.00	82.00	30.00	30.00
(p25, p75)	(47.00, 53.00)	(78.00, 86.00)	(26.50, 33.00)	(23.00, 39.00)

**, at the 0.01 level (two-tailed), the correlation is significant.

### 3.4 The mediating effect of self-compassion

To verify the mediating role of self-compassion between transition shock and moral sensitivity, Model 4 in Hayes’PROCESS v4.2 macro program was used to establish a mediation model. The results, presented in Table 3, showed that transition shock could significantly negatively predict moral sensitivity (*β* = −0.213, *p* = 0.001) and self-compassion (*β* = −0.716, *p* < 0.001), supporting Hypotheses 1 and 2; while self-compassion could significantly positively predict moral sensitivity (*β* = 0.221, *p* < 0.001),supporting Hypothesis 3. The mediation model was further tested, and the results are shown in Table 4. The direct effect of transition shock on moral sensitivity was significant [*B* = −0.213, *p* < 0.001, 95% CI (−0.335, −0.091)], and the indirect effect through self-compassion was also significant [*B* = −0.158, *p* < 0.001, 95% CI (−0.241, −0.089)]. The mediating effect accounted for 42.59% of the total effect, thereby supporting Hypothesis 4.

**Table 3:** Regression results of the mediating effect of self-compassion.

Dependent variable	Independent variable	*β*	*SE*	*t*	*p*	*R^2^*	*F*
Self-compassion	Transition shock	−0.716	0.108	−6.632	0.000	0.125	43.985
Moral sensitivity	Transition shock	−0.213	0.062	−3.444	0.001	0.234	46.655
	Self-compassion	0.221	0.031	7.221	0.000		

**Table 4:** Mediating effect values of self-compassion between transition shock and moral sensitivity.

Project	Effect value	*SE*	LLCI	ULCI	Relative effect value (%)
Direct effect	−0.213	0.062	−0.335	−0.091	57.41%
Indirect effect	−0.158	0.040	−0.241	−0.089	42.59%
Total effect	−0.371	0.062	−0.494	−0.248	100.00%

### 3.5 The moderating role of calling

**Figure 2:**
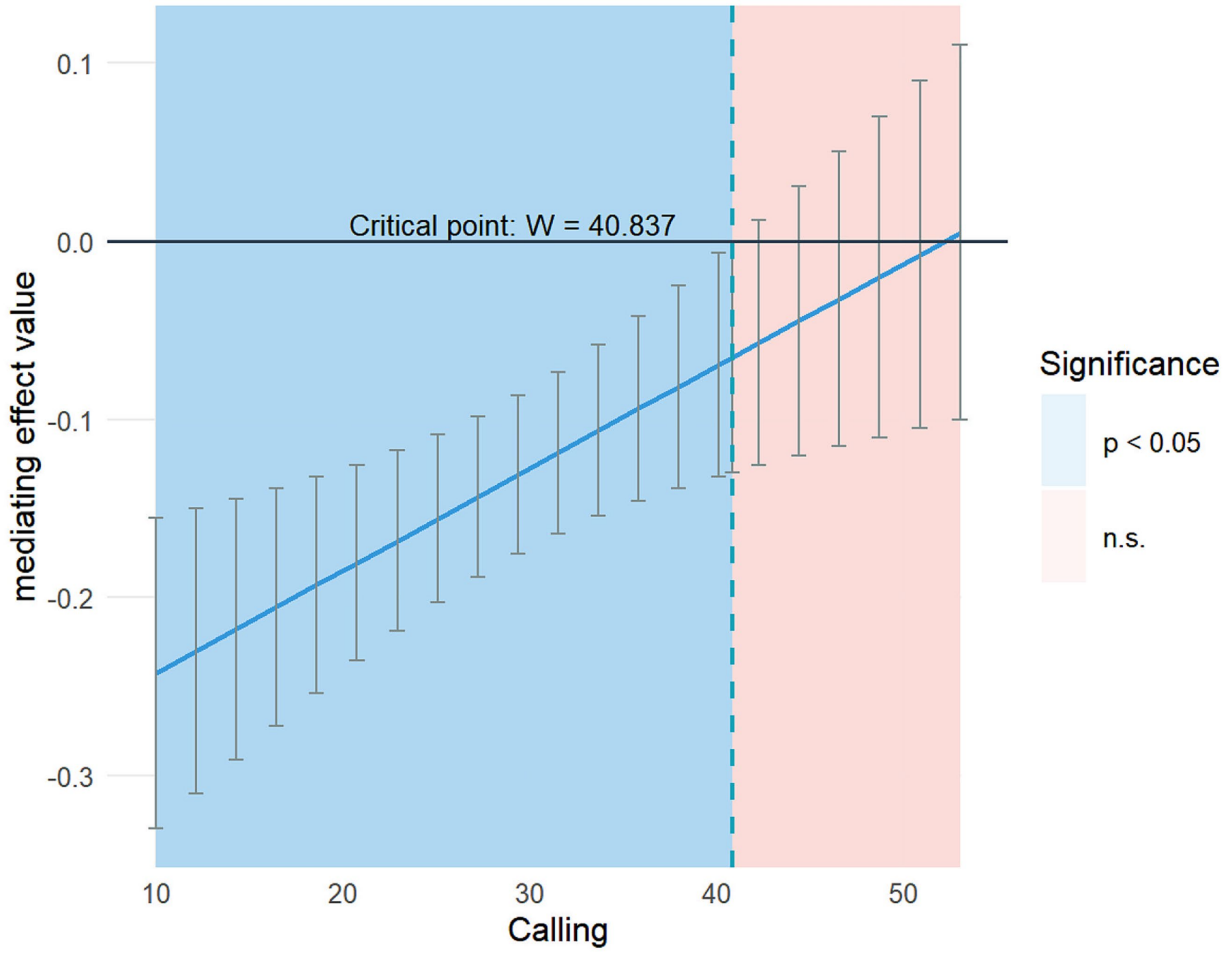
Changes in the mediating effect value of self-compassion with calling.

To further verify the moderated mediation effect of calling in the mediating model, relevant regression analyses were conducted for each variable. The results are shown in Table 5. significantly moderated the effect of transition shock on self-compassion (*B* = 0.026, *p* < 0.001). To further verify the hypothesis, Model 7 in the PROCESS v4.2 macro program was used to examine the moderated mediation model. The results are shown in Table 6. As calling increased, the mediating effect value of self-compassion between transition shock and moral sensitivity gradually decreased. To further clarify the critical value of the moderating effect of calling, RStudio was used toexamine and plot the variation in the mediating effect value of self-compassion with calling, as shown in Figure 2. The critical value of the moderating effect of calling was 40.837.

**Table 5:** Analysis and verification of the moderating effect of calling.

Variable	Equation 1 Outcome indicator (M)			Equation 2 Outcome indicator (Y)		
	*B*	*β*	*t*	*B*	*β*	*t*
X	−1.367^***^	0.286	−4.770	−0.213^***^	0.062	−3.444
M	−1.041^*^	0.466	−2.236	0.221^***^	0.031	7.221
X*W	0.026^**^	0.009	2.841	—	—	—
*R^2^*	0.239			0.234		
*F*	31.952^***^			46.655^***^		

X: transition shock.

M: self-compassion.

W: calling.

Y: moral sensitivity.

^*^p < 0.05, ^**^p < 0.01, ^***^p < 0.001.

**Table 6:** The impact of different levels of adjustment on the mediation effect size in the mediation model.

Level of professional mission	Mediating effect size	Boot SE	Boot LLCI	Boot ULCI
M − SD	−0.183	0.048	−0.283	−0.096
Mean	−0.120	0.033	−0.191	−0.062
M + SD	−0.058	0.037	−0.138	0.008

**Figure 3:**
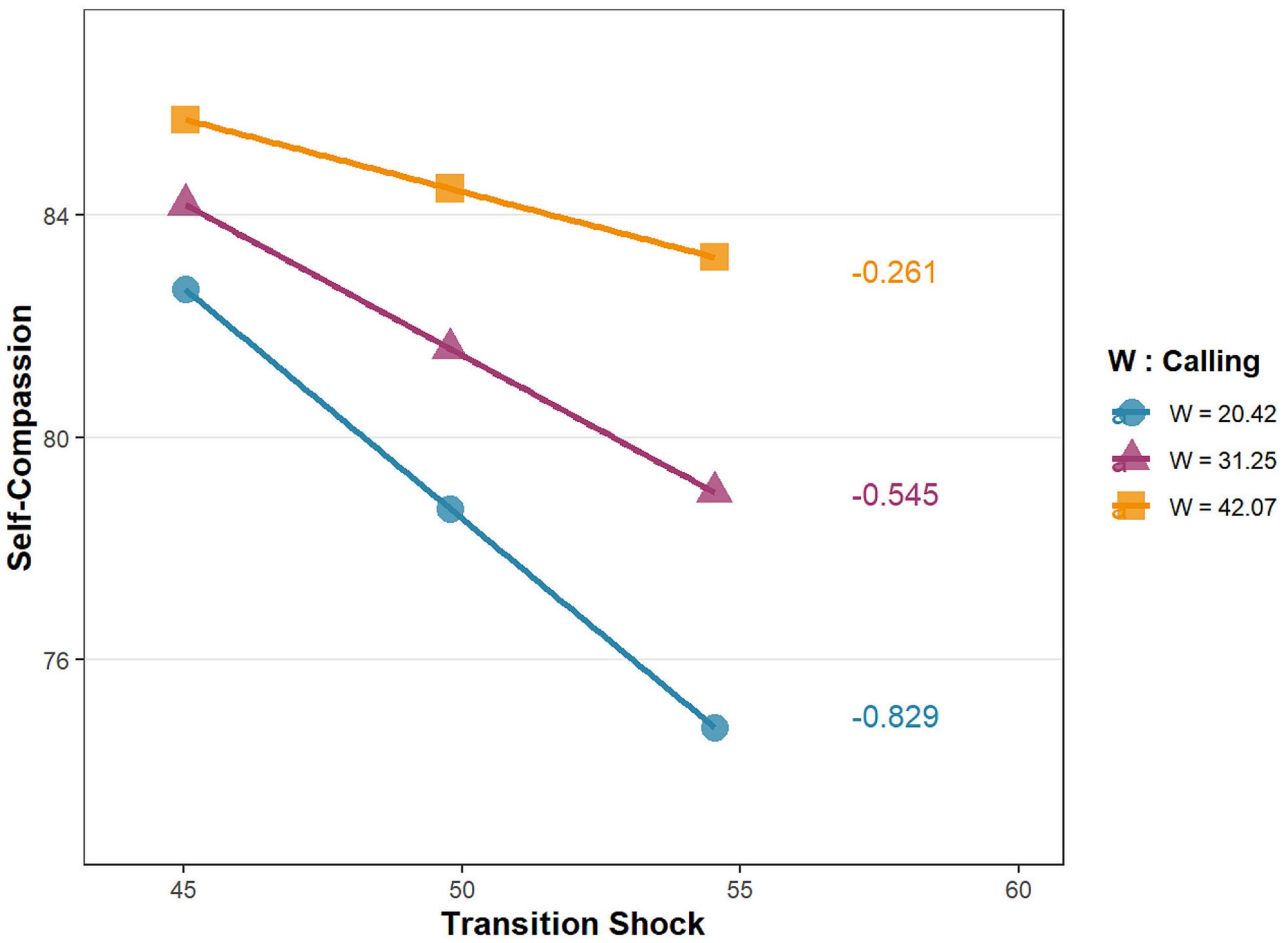
Simple slope plot of calling moderating the relationship between transition shock and self-compassion.

To visually examine the moderating effect of calling on the first stage of the mediating effect model, simple slope test was conducted. Calling was divided into low, medium, and high levels, corresponding to M-SD, M, and M + SD, respectively. As shown in Figure 3, when calling was at a low level, transition shock could significantly negatively predict self-compassion (*βsimple* = −0.829, *p* < 0.001); when calling was at a medium level, transition shock could significantly negatively predict self-compassion (*βsimple* = −0.545, *p* < 0.001); when calling was at a high level, the negative predictive effect of transition shock on self-compassion was no longer statistically significant (*βsimple* = −0.261, *p* = 0.102). These results indicate that as calling increases, the negative predictive effect of transition shock on self-compassion was gradually weakened, suggesting that calling acted as a positive moderator in the impact of transition shock on self-compassion. Hypothesis 5 was supported.

## 4 Discussion

The study found that the median score of moral sensitivity was 30, indicating that nursing interns had a low level of moral sensitivity. The score of moral sensitivity in this study was significantly lower than that reported in the study by Guo et al. (42). The main reason for this difference was the variation in research subjects: the latter investigated registered nurses with more than 1 year of work experience, confirming that there was a significant difference in moral sensitivity between nursing interns and registered nurses. The median score of transition shock among nursing interns was 50, indicating a relatively high level, which was broadly consistent with the findings of Zhao et al. (43). This suggests that nursing interns experience severe transition shock. High transition shock can reduce work efficiency, deplete psychological resources, and hinder the transition to the role of a professional nurse, which warrants attention from nursing managers (44). The median score of self-compassion was 82, at moderate-high level, slightly lower than that reported by Wang et al. (45). The reason is that the latter surveyed nursing students in school who had not entered clinical internships and were not affected by transition shock, so they had higher self-compassion scores. This finding also indirectly confirms the negative impact of transition shock on self-compassion. The median score of calling was 30, broadly consistent with the study by Chen et al. (46) among nursing students not yet in clinical practice, with both groups at a moderate level per the scale’s scoring criteria. This indicates that educational institutions lack effective cultivation of calling among nursing students. Some nurses have limited cognition of the nursing profession, failing to fully understand the sacred mission it bears in safeguarding life and health, and only regard it as a technical job of “carrying out procedures and completing tasks,” ignoring the humanistic care and noble value inherent in the profession (47).

The results confirmed that the higher the level of transition shock among nursing interns, the lower their moral sensitivity scores, supporting Hypothesis 1. This finding was highly consistent with the findings of Ryus et al. (48) that stress impairs the ability to perceive ethical issues. From clinical practice perspective, nursing interns are in a critical transition period and face stressors and challenges such as “theory-practice gap” and “high pressure from clinical skill performance” (44). When confronted with these shocks, their cognitive resources were prioritized to meet core needs—such as how to perform tasks assigned by preceptors and how to avoid operational errors—thereby reducing their ability to perceive ethical cues including patient privacy and patients’ right to informed consent. Fundamentally, this result supported the core proposition of the Conservation of Resources Theory: as a resource-depleting event, transition shock caused nursing interns to shift attention away from non-urgent needs (e.g., ethical perception). This established a mechanism whereby transition shock induces resource depletion, which in turn reduces moral sensitivity.

The results showed that transition shock was significantly negatively correlated with self-compassion among nursing interns—specifically, the stronger the transition shock, the weaker the interns’ capacity for self-kind acceptance and mindful awareness—supporting Hypothesis 2. This finding could be interpreted from the psychological characteristics of nursing interns. On one hand, the frustrating experiences associated with transition shock (such as criticism from preceptors and misunderstandings by patients) were likely to trigger self-criticism, which contradicts the core dimension of self-compassion, namely “self-kindness.” On the other hand, “emotional exhaustion” caused by transition shock would weaken individuals’ mindfulness capacity. Combined with the Conservation of Resources Theory (COR) (16) and the Self-Compassion Theory (17), an analysis from a clinical practice perspective reveals that transition shock, as a profession-specific stressor, directly impairs the self-compassion capacity of nursing interns. This impairment in turn exacerbated difficulties in transition adaptation, creating a vicious cycle in which transition shock leads to decreased self-compassion, which further increases adaptation challenges. This suggests that self-compassion may be a key buffering variable to break this cycle.

The study confirmed that the higher the level of self-compassion among nursing interns, the stronger their moral sensitivity, supporting Hypothesis 3. This provided new empirical evidence for the viewpoint that positive psychological resources foster ethical literacy (49). An analysis from the core dimensions of self-compassion revealed three key mechanisms: First, “self-kindness” helped nursing interns reduce self-criticism, enabling them to allocate more psychological resources to patients’ ethical needs (50). Second, “common humanity” allowed interns to recognize that difficulties during the transition period are shared experiences among all novice nurses, preventing self-isolation and encouraging them to proactively attend to patients’ ethical appeals (51). Third, “mindful awareness” enhanced interns’ ability to detect ethical situational cues (52). This result indicated that in addition to ethical knowledge training, efforts to cultivate psychological resources among nursing interns should also be strengthened. It suggested that self-compassion is not merely a self-regulatory tool; rather, it can act as an intrinsic driver force for the development of moral sensitivity among nursing, by enhancing psychological resilience, optimizing interpersonal perception, and strengthening ethical awareness.

Bootstrap testing results showed that the mediating effect of self-compassion between transition shock and moral sensitivity was significant, accounting for 42.59% of the total effect, which supported Hypothesis 4. Specifically, transition shock could directly impair the moral sensitivity of nursing interns, and could also indirectly weaken their moral sensitivity by reducing self-compassion. In practice, when nursing interns face high transition shock, the resource-buffering effect of self-compassion is impaired. On one hand, the decline in self-compassion makes it difficult for interns to alleviate anxiety through self-kindness, thereby failing to detect ethical cues (53). On the other hand, the lack of mindful awareness leads interns to experience emotional exhaustion (54), diminishing their ability to perceive patients’ ethical needs. Conversely, if nursing interns have high levels of self-compassion, even in the face of transition shock, they can maintain sensitivity to ethical situations by accepting their limitations and being mindful of their emotional states, thereby buffering the negative impact of transition shock. Verification of this mediating mechanism provides a targeted focus for the design of subsequent intervention programs. It indicated that improving the self-compassion of nursing interns can serve as a dual strategy that both alleviates transition shock and preserves moral sensitivity.

The moderating effect analysis showed that calling significantly moderated the relationship between transition shock and self-compassion: as calling increased, the negative impact of transition shock on self-compassion gradually weakened, supporting Hypothesis 5. This result was consistent with the core tenet of the Meaning Maintenance Model (24)—that is, as a source of meaning, calling helps nursing interns reconstruct their appraisal of difficulties when confronted with transition shock. Interns with high calling tended to view clinical procedural errors and criticism from preceptors as necessary experiences for becoming qualified nurses, rather than threats to their self-worth, and thus were more likely to maintain self-kindness. Meanwhile, recognition of the value of the nursing profession endows interns with psychological energy to cope with stress, reducing the loss of mindfulness caused by shock (55). This study confirmed that calling acts as a protective factor, creating a psychological buffer for nursing interns under high transition shock. For instance, when both face patient complaints, interns with high calling were more likely to reflect on how to improve their communication methods (self-compassion-oriented), whereas those with low calling tended to lapse into self-negation such as “I am not suitable for nursing work” (self-criticism-oriented).

## 5 Practical implications and recommendations

### 5.1 School level

Establish a pre-internship training system guided by both self-compassion and calling. Specific measures are as follows: Offer specialized self-compassion training courses: Develop mindfulness training modules integrated with nursing internship contexts to help nursing interns recognize and regulate their emotions and reduce emotional exhaustion. Guide interns to attribute clinical operational errors to insufficient experience rather than innate incompetence, thereby fostering their self-compassion capacity at the root. Enhance calling cultivation: Help interns intuitively feel the value of the nursing profession by sharing cases of outstanding nurses and patient gratitude stories, so as to alleviate career confusion. Simulate clinical dilemma situations before the internship to guide interns to reconstruct positive cognitive appraisals of meaning.

### 5.2 Hospital level

Optimize clinical support and preceptorship models for nursing interns. Specific measures are as follows: Develop preceptorship plans based on interns’ transition shock levels. For example, interns with high transition shock should receive more emotional support, and the pressure from clinical operational tasks should be reduced in the early stages of their. Implement a dual-mentor system comprising clinical preceptors and career development mentors. Career development mentors hold regular discussions on career planning with interns to reinforce their calling.

### 5.3 Individual level

Guide nursing interns to proactively activate psychological resources. Specific measures are as follows: Encourage interns to use the “Three Self-Compassion Questions” (15) to cope with transition shock: “What do I need right now?” “How would I comfort a friend in this situation?” “Do other interns also encounter this difficulty?” This helps translate self-compassion into daily coping strategies. Advise interns to develop “career meaning cards” and review them when confronting transition shock, so as to buffer the decline in self-compassion by activating meaning.

## 6 Study limitations

First, this study adopted convenience sampling and online questionnaires, which may be subject to selective response bias. Interns experiencing severe transition shock are more likely to refuse participation, resulting in underrepresentation of cases with high transition shock in the sample and potential underestimation of the true magnitude of associations between variables. Second, transition shock, self-compassion, and moral sensitivity among nursing interns all exhibit stage-specific characteristics and may change with the duration of the internship. The cross-sectional design of this study pooled samples from different internship stages for analysis, which to a certain extent obscures differences in the magnitude of mediating and moderating effects across stages. Future studies could expand the sample size and conduct subgroup analyses accordingly. Third, the sample was only recruited from 6 hospitals in one province, lacking sufficient representativeness in terms of geography and hospital levels, which may limit the generalizability of the results. Future research could expand the research settings and populations to verify the generalizability of the findings. Fourth, the measurement tools relied on self-report scales, which are inherently subjective and prone to measurement bias. In future studies, the accuracy of measurement could be improved by integrating methods such as situational simulation assessments. Fifth, this study did not incorporate potential moderating variables (e.g., preceptorship quality). These variables may jointly influence the relationship between transition shock and self-compassion alongside calling, and thus deserve further in-depth exploration in future research.

## 7 Conclusion

This study empirically confirmed that transition shock among nursing interns negatively affects moral sensitivity and self-compassion, in which self-compassion plays a mediating role; furthermore, calling serves to buffer the negative impact of transition shock on self-compassion. This result not only enriches the theoretical research on the relationship between nursing internship transition and ethical literacy but also provides actionable intervention targets for educational institutions and hospitals to develop “psychological support and ethical cultivation programs for nursing interns” in practice. By fostering self-compassion and enhancing calling, nursing interns can be helped to better cope with transition shock, protect the development of their moral sensitivity, and lay a solid foundation for their development into qualified nurses equipped with empathy and ethical literacy.

## Data Availability

The original contributions presented in the study are included in the article/supplementary material, further inquiries can be directed to the corresponding author.
